# Increased susceptibility to Chrysanthemum Yellows phytoplasma infection in *Atcals7ko* plants is accompanied by enhanced expression of carbohydrate transporters

**DOI:** 10.1007/s00425-022-03954-8

**Published:** 2022-07-17

**Authors:** Chiara Bernardini, Simonetta Santi, Giovanni Mian, Amit Levy, Sara Buoso, Joon Hyuk Suh, Yu Wang, Christopher Vincent, Aart J. E. van Bel, Rita Musetti

**Affiliations:** 1 Department of Agricultural, Food, Environmental and Animal Sciences, University of Udine, via delle Scienze, 206, 33100 Udine, Italy; 2 Department of Plant Pathology, Citrus Research and Education Center, University of Florida, 700 Experiment Station Rd, Lake Alfred, FL 33850 USA; 3 Department of Food Science and Human Nutrition, Citrus Research and Education Center, University of Florida, 700 Experiment Station Rd, Lake Alfred, FL 33850 USA; 4 Horticultural Sciences Department, Citrus Research and Education Center, University of Florida, 700 Experiment Station Rd, Lake Alfred, FL 33850 USA; 5 Institute of Phytopathology, Justus-Liebig University, Heinrich-Buff-Ring 26–32, 35392 Giessen, Germany; 6 Department of Land, Environment, Agriculture and Forestry (TESAF), Università di Padova, via dell’ Università, 16, 35020 Legnaro, PD Italy

**Keywords:** *Arabidopsis*, Callose, Defence, Phloem, Phytoplasma, Sugar metabolism, Transport

## Abstract

**Main conclusion:**

**Loss of CALS7 appears to confer increased susceptibility to phytoplasma infection in Arabidopsis, altering expression of genes involved in sugar metabolism and membrane transport**.

**Abstract:**

Callose deposition around sieve pores, under control of callose synthase 7 (*CALS7*), has been interpreted as a mechanical response to limit pathogen spread in phytoplasma-infected plants. Wild-type and *Atcals7ko* mutants were, therefore, employed to unveil the mode of involvement of *CALS7* in the plant’s response to phytoplasma infection. The fresh weights of healthy and CY-(Chrysanthemum Yellows) phytoplasma-infected Arabidopsis wild type and mutant plants indicated two superimposed effects of the absence of *CALS7*: a partial impairment of photo-assimilate transport and a stimulated phytoplasma proliferation as illustrated by a significantly increased phytoplasma titre in *Atcal7ko* mutants. Further studies solely dealt with the effects of *CALS7* absence on phytoplasma growth. Phytoplasma infection affected sieve-element substructure to a larger extent in mutants than in wild-type plants, which was also true for the levels of some free carbohydrates. Moreover, infection induced a similar upregulation of gene expression of enzymes involved in sucrose cleavage (*AtSUS5, AtSUS6*) and transmembrane transport (*AtSWEET11*) in mutants and wild-type plants, but an increased gene expression of carbohydrate transmembrane transporters (*AtSWEET12*, *AtSTP13*, *AtSUC3*) in infected mutants only. It remains still unclear how the absence of AtCALS7 leads to gene upregulation and how an increased intercellular mobility of carbohydrates and possibly effectors contributes to a higher susceptibility. It is also unclear if modified sieve-pore structures in mutants allow a better spread of phytoplasmas giving rise to higher titre.

**Supplementary Information:**

The online version contains supplementary material available at 10.1007/s00425-022-03954-8.

## Introduction

Phytoplasmas are wall-less, pleomorphic plant pathogens belonging to the class *Mollicutes*. Their presence is confined to the sieve elements (SEs) of host plants (van Bel and Musetti 2019) or to the body of phloem-feeding insect vectors (Alma et al. 2019). Phytoplasmas cause serious yield losses and affect the quality of crops of economic interest (Namba et al. 2019). Profound phytoplasma-induced alterations in transcriptome and proteome (Cao et al. 2017, 2019), and in the phytohormone balance (Dermastia 2019; Bernardini et al. 2020) are reflected by a vast range of symptoms, such as witches’-brooms, leaf chlorosis, virescence, phyllody, and floral abortion (Ermacora and Osler 2019), often leading to sterility and unproductiveness of the host plant (Namba et al. 2019).

Plants react to phytoplasma invasion by mechanical occlusion of sieve pores, which is probably due to a fast plugging by specialized proteins (Will et al. 2013; Pagliari et al. 2017; Walker et al. 2022), followed by a slower constriction due to additional deposition of callose around the pores (Musetti et al. 2010, 2013; Santi et al. 2013). In *Arabidopsis thaliana*, 12 genes (*CALS1-12*) encoding for callose synthase (CALS) enzymes have been found (Xie and Hong 2011). The diverse CALS-gene expression patterns throughout the plant are indicative of differential local impact of callose synthesis (Ellinger and Voigt 2014). As for the phloem, Barratt et al. (2011) and Xie et al. (2011) demonstrated that *AtCALS7* encodes for callose synthase 7 (AtCALS7), the enzyme responsible for callose deposition around sieve pores. AtCALS7 also regulates sieve-plate development during phloem differentiation, which is an important determinant of mass flow in mature sieve tubes, and, hence, influences the carbohydrate availability for sinks and, therefore, the plant growth (Xie et al. 2011).

Synthesis of the cell-wall polymer callose (1,3-β-d-glucan) requires sucrose units that are cleaved by sucrose synthases (SUSs) into fructose and UDP-glucose, the substrate for callose synthase. Arabidopsis callose synthase proteins contain multiple transmembrane domains that are clustered into two regions (N-terminal and C-terminal), leaving a large hydrophilic central loop that faces the cytoplasm. This loop contains the putative catalytic domain, which has been further subdivided into two domains: the UDP-glucose binding domain and the glycosyltransferase domain (Wang et al. 2022). In the sieve tubes of Arabidopsis, two membrane-bound sucrose synthase isozymes, AtSUS5 and AtSUS6 (Barratt et al. 2009), associated with AtCALS7 form a unique enzyme complex (Bieniawska et al. 2007; Ruan 2014; Stein and Granot 2019).

There are various levels at which phytoplasmas and CALS7 might act and interact with major consequences for carbohydrate household. Absence of CALS7 as a strong determinant of proper sieve-pore development may modify carbohydrate household as indicated by the lower growth rates of *Atcals7ko* mutants (Xie et al. 2011). Furthermore, sucrose content of sieve-tube sap might be affected by carbohydrate consumption by dividing and growing phytoplasmas. Activation or increased expression of CALS7 in mature sieve tubes by wounding (Barratt et al. 2011) or pathogenic effectors elicit callose-mediated sieve-pore constriction that blocks the fluorochrome-labelled phloem stream (Musetti et al. 2013; Pagliari et al. 2017). Callose synthesis requires glucose building blocks that are recruited from sucrose molecules in the phloem stream by intervention of SUS5 and SUS6 (Bieniawska et al. 2007; Barratt et al. 2009; Ruan 2014; Stein and Granot 2019).

As infected plants show reduced, but still appreciable growth rates (Pagliari et al. 2017), the constricted sieve pores may not be fully occluded for photosynthate translocation. As an alternative explanation for the photosynthate delivery to sinks, photosynthates may circumvent the occluded sieve pores using the hop-on-hop-off release/retrieval mechanisms along the sieve tubes (De Schepper et al. 2013; Stadler 2021; van Bel 2021). The rates of release and retrieval are controlled by transporters such as STPs, SUTs and SWEETs along the pathway (van Bel 2021). According to this concept, CALS7-mediated sieve-tube occlusion would coincide with an intensified CC/SE plasma membrane transport of sugars dependent on an upregulated expression of genes encoding sugar transporters to maintain phloem transport. Aim of the present work was to investigate whether and to what extent the sieve-element specific AtCALS7 interferes with plant sugar translocation and metabolism in response to phytoplasma infection. Wild-type and *Atcals7ko* [a mutant line in which the gene encoding for AtCALS7 was silenced (Barratt et al. 2011)] Arabidopsis plants were infected with Chrysanthemum Yellows (CY)-phytoplasma, and growth rates, phytoplasma titres, plant morphology, sieve-tube substructure, carbohydrate composition were compared. Moreover, the expression of several carbohydrate-handling genes was evaluated, focussing on those encoding transporters or effluxers located in the phloem. The transporters in question are: AtSUC2/SUT1 and AtSUC3/SUT2 (hereafter referred to as AtSUC2 and AtSUC3), which are sucrose proton cotransporters, located respectively in CCs and in SEs (Stadler and Sauer 1996; Meyer et al. 2004); AtSWEET11 and AtSWEET12 being sugar transport facilitators from the Sugar Will Eventually Be Exported Transporters family (SWEETs) localized to the plasma membrane of parenchyma cells in the phloem-loading zone (Chen et al. 2012; Le Hir et al. 2015); AtSTP13/MSS1, a member of the Sugar Transporter Protein (STP) family, is a vascular high affinity, hexose proton symporter mediating the uptake/retrieval of hexoses from the apoplastic space (Büttner 2010) across the plasma membrane (Zakhartsev et al. 2016; Julius et al. 2017) and being involved in the plant response to pathogens (Yamada et al. 2016; Skoppek et al. 2022). Finally, the cell-wall invertases AtCWINV1 and AtCWINV6 present the first in almost all plant tissues, and the second in the leaves (Sherson et al. 2003), and involved in the irreversible cleavage of sucrose into glucose and fructose in the apoplast.

## Materials and methods

### Plants and insect vectors

Seeds of wild type and *Atcals7ko* (SALK_048921) lines of *Arabidopsis thaliana* ecotype Col0 were provided by the Nottingham Arabidopsis Stock Centre (NASC). Sixty four plants were grown in a climate chamber for 40 days on 5:1 mixture of soil substrate and perlite and fertilized twice a month with an N–P–K liquid fertilizer, as described previously (Pagliari et al. 2017), 32 plants under short-day light conditions (9 h L/15 h D) at 18–20 °C, and 32 under long-day light conditions (14 h L/10 h D), at 23 °C to develop the floral stem for the transport speed experiment.

Healthy colonies of *Macrosteles quadripunctulatus* were reared on *Avena sativa* in vented plexiglass cages in greenhouse (temperature of 20–22 °C and short-day conditions 9 h L/15 h D), according to Bosco et al. (1997). The last instar nymphs were transferred to *Chrysanthemum carinatum* plants infected with Chrysanthemum Yellows (CY) phytoplasma (Lee et al. 2003), a strain related to ‘*Candidatus* Phytoplasma asteris’ (‘*Ca*. P. asteris’, 16SrI-B subgroup), as the source of inoculum for a 7 day-phytoplasma acquisition-access period (AAP). After a latent period (LP) of 18 days, insects were transferred to 40-day-old Arabidopsis plants for the 7 day-inoculation access period (IAP).

Eight wild type and eight *Atcals7ko* plants were exposed to 3 infectious *M. quadripunctulatus* individuals (CY-infected plants). In addition, 16 plants from both lines (8 + 8) were exposed to 3 healthy vectors as a control (healthy plants). Healthy leafhoppers were collected from healthy colonies and were of the same age as the infected ones. When symptoms on the infected plants grown under short-day conditions were clearly visible, i.e. 26 days post inoculation (Pacifico et al. 2015; Pagliari et al. 2017), midribs from the leaves of the third rosette node (source leaves) were collected. For phytoplasma detection and gene expression analyses, collected samples were immediately frozen in liquid nitrogen and stored at − 80 °C until use. For carbohydrate analyses, midribs were immediately frozen in liquid nitrogen, freeze-dried and then stored at − 80 °C until use.

### Symptom description

Symptom development was observed in healthy and CY-infected plants of both lines, from the end of the inoculation period to the time of tissue harvest for different analyses, i.e. when plants were ca. 70 day-old. To evaluate the phenotypic differences between the two lines and the differences due to phytoplasma infection, the rosette fresh weight was measured in plants grown under short-day conditions using 8 healthy and 8 CY-infected plants per line. The day before sampling, soil was saturated with water. Rosettes were then cut at the plant collar level and the fresh weight of each biological replicate was measured. Moreover, the length of the floral stalk was measured in plants grown under long-day light conditions (see above), using 8 healthy and 8 CY-infected plants per line. Statistical analyses were performed using RStudio software Version 1.1.456 (RStudio Team 2020, Boston, MA, USA). The normal distribution was checked with the Shapiro–Wilk test. Significant differences among the group means were determined with a two-way ANOVA and post-hoc comparisons between all groups were made with Tukey’s test with *P* < 0.05.

### Phytoplasma detection and quantification

To check the phytoplasma titre in wild type and *Atcals7ko* CY-infected plants, genomic DNA was extracted from 200 mg of fresh leaf midrib tissue, according to Doyle and Doyle protocol (1990) modified by Martini et al. (2009). The ribosomal protein gene *rplV* (*rpl22*) was chosen as a target for the amplification of CY-phytoplasma DNA using the primer pair rp(I-B)F2/rp(I-B)R2 (Lee et al. 2003; Pagliari et al. 2017) and a CFX96 real-time PCR detection system (Bio-Rad Laboratories, Richmond, CA, USA). A standard curve was established by tenfold serial dilutions of a plasmid DNA containing the 1260 bp ribosomal protein fragment from CY phytoplasma, amplified with the primer pair rpF1C/rp(I)R1A (Pagliari et al. 2017). Real-time PCR mixture and cycling conditions were previously described (Pagliari et al. 2017). The phytoplasma titre was expressed as the number of CY-phytoplasma genome units (GUs) per mg of fresh leaf sample to normalize the data. Statistical significance of the quantitative differences between phytoplasma populations was calculated by analysis of three replicates of 8 plants per line. Statistical analyses were performed using RStudio software version 1.1.456 (2009–2018 RStudio). The normal distribution was checked with Shapiro–Wilk test. Significant differences among the means were determined using the Kruskal–Wallis non-parametric test with *P* < 0.05.

### Phloem transport linear speed measurement

For phloem transport experiments, Arabidopsis plants were grown as reported above, under long-day light conditions (14 h L/10 h D), at 23 °C. Sixty-day-old healthy plants of both lines, were used for the measurement. Two x-ray photomultiplier tubes, 5.6 cm in diameter, (St. Gobain, Malvern, PA, USA) were placed along the floral stem of both wild type and *Atcals7ko* plants, leaving a 2-cm-long buffer zone between the detectors, while a third detector monitored the signal from the rosette (Supplementary File S1).

Each detector was connected to an M4612 12-channel counter and counts of x-rays were logged with the manufacturer’s software (Ludlum Measurements, Sweetwater, TX, USA). Plants were placed on a lead layer having a receptacle, under the same day length used for growing. After at least 8 h of background measurement, 600 μl of NaH^14^CO_3_ solution (specific activity: 40–60 mCi (1.48–2.22 GBq)/mmol) were injected into the receptacle. Immediately thereafter, 1000 µl of a saturated citric acid solution was also injected into the receptacle. Plants were tightly closed in a plastic bag, and were allowed to assimilate ^14^CO_2_ for 2 h. Then the bag was removed and the remaining gaseous radiolabelled isotope was flushed via the fume hood. The x-ray detectors monitored the x-ray counts from the plant tissue every minute for 72 h. The distance between the detectors was measured at the end of the experiment.

The data of the counts from each detector were handled with the < *nmle* > package in Rstudio (Pinheiro et al. 2018). In accordance with previously published methods, the Rstudio script was written to create a logistic function, and to fit the logistic function to the data with time on the x-axis and the recorded counts on the y-axis (Vincent et al. 2019). Xmid, which represents the time at the half-height of the logistic curve or the average time of arrival of ^14^C in the phloem tissue near the x-ray detector, was calculated for each detector placed along the stem. The speed of translocation was calculated by dividing the distance between the middle of the two detectors (in cm) and the difference between the Xmid timepoints from the detectors (in h). Both infected lines failed to normally develop flowering stalks: stalk was very short in infected wild-type plants and absent in infected *Atcals7ko* (Fig. 2c). For these reasons, it was not possible to calculate and compare sugar translocation speed in the two infected Arabidopsis lines.

Four healthy plants per line were used for the evaluation of the phloem transport linear speed. For both wild type and *Atcals7ko* healthy plants, 4 measurements were carried out, pairing 1 wild type and 1 *Atcals7ko* together in each labelling.

### Gene expression analyses

Total RNA was extracted from 100 mg of leaf midrib powder, from 5 plants for the four experimental conditions, obtained by grinding in liquid nitrogen and using a Spectrum^™^ Plant Total RNA kit (Sigma-Aldrich, Merck, Darmstadt, Germany) according to the manufacturer’s instructions. The RNA reverse-transcription was carried out using a QuantiTectReverse Transcription Kit (Qiagen, Hilden, Germany) following the manufacturer’s instructions. The expression of the genes was analysed in healthy and CY-infected midribs by real-time experiments, performed on a CFX96 real-time PCR detection system (Bio-Rad Laboratories). The reference gene was selected by comparing the *AtUBC9* (ubiquitin conjugating enzyme 9), *AtTIP41* (TIP41-like family protein), *AtSAND* (SAND family protein), and *AtUBQ10* (polyubiquitin 10) gene expression. The gene stability values (M values) were calculated according to the geNorm programme (Pagliari et al. 2017). *AtUBC9* gene was the most stably expressed gene and so the most suitable as a reference gene (*M* = 0.44). SsoFast EvaGreen Supermix 2× (Bio-Rad Laboratories) and cDNA obtained from 5 ng of RNA and specific primers (Supplementary Table S1) were used in a total volume of 10 μl. Under these conditions, the primer pair efficiency was evaluated as described by Pfaffl (2001) using standard curves of different dilutions of pooled cDNA. PCR was performed as described in Pagliari et al. (2017), with three technical repeats. A mean normalized expression (MNE) for each gene of interest (Muller et al. 2002) was calculated by normalizing its mean expression level to the level of the UBC9 gene. Five individuals were used for the gene MNE determination. Statistical analyses were performed using RStudio software Version 1.1.456 (2009–2018 RStudio). The normal distribution was checked with Shapiro–Wilk test. Significant differences among the means were determined by a two-way ANOVA and post-hoc comparisons between all groups were made with Tukey’s test with *P* < 0.05.

### Transmission electron microscopy

To observe phloem ultrastructure in the midrib, samples were prepared for microscopic analyses as reported by Pagliari et al. (2016). For each condition, at least five segments (10 mm in length) of the mature leaf midrib from 5 individuals were submerged in MES buffer (10 mM NaOH-2-(*N*-morpholino) ethanesulfonic acid, 2 mM CaCl_2_, 1 mM MgCl_2_, 0.5 mM KCl and 200 mM mannitol, pH 5.7) for 2 h at room temperature. Samples were fixed in a solution of 3% paraformaldehyde and 4% glutaraldehyde for 6 h, the solution was refreshed every 30 min. After rinsing, samples were post-fixed overnight with 2% (w/v) OsO_4_, dehydrated with an ethanol gradient and transferred to pure propylene oxide. Midrib segments were then embedded in Epon/Araldite epoxy resin (Electron Microscopy Sciences, Fort Washington, PA, USA). Ultra-thin sections were collected on uncoated copper grids, stained with UAR-EMS (uranyl acetate replacement stain, Electron Microscopy Sciences) and then observed under a PHILIPS CM 10 (FEI, Eindhoven, The Netherlands) transmission electron microscope (TEM), operated at 80 kV, and equipped with a Megaview G3 CCD camera (EMSIS GmbH, Münster, Germany). Five non-serial cross-sections from each sample were analysed.

### Sugar quantification

Authentic standards of sugars (rhamnose, arabinose, fructose, glucose, maltose, sucrose, and melibiose) and sugar alcohols (glycerol, myo-inositol, arabitol, and sorbitol) were purchased from Sigma-Aldrich. Internal standards including fructose-^13^C6 (for sugars) and sorbitol-^13^C6 (for sugar alcohols) were obtained from Toronto Research Chemicals (Toronto, ON, Canada) and Sigma-Aldrich (St. Louis, MO, USA), respectively. Water, acetonitrile, methanol, and formic acid were of LC–MS grade, and were purchased from Fisher Scientific (Fair Lawn, NJ, USA). Stock solutions of each analyte and internal standard were prepared at a concentration of 10,000 μg/ml in water, or methanol. Working standard solutions were prepared by diluting and mixing each stock solutions with 90% methanol (water/methanol, 10/90, v/v). The stock and working solutions were stored at − 80 °C. Freeze-dried leaf midribs were stored at − 80 °C until use. Ten milligram of ground samples treated with 0.05 ml of internal standard solution (50 μg/ml sorbitol-13C6 and 200 μg/ml fructose-13C6 in 90% acetonitrile (water/acetonitrile, 10/90, v/v) was extracted with 0.95 ml of 90% acetonitrile (water/acetonitrile, 10/90, v/v) (total volume: 1 ml) by ultra-sonication for 30 min, followed by agitation for 30 min. After centrifugation (20,000 g, 5 min, 4 °C), supernatant was further filtered through 0.22 μm nylon filter, and was injected into LC–MS/MS for analysis. The extraction was performed in triplicate using 4 biological replicates. Data obtained by analyses, were handled using RStudio software. Normality of the data was checked with Shapiro–Wilk test, outliers were removed, and data were normalized, where necessary, with Box-Cox transformation. A two-way analysis was performed followed by post-hoc pairwise comparison of all groups with Tukey’s test, with *P* < 0.05.

## Results

### Phenotype, symptom appearance, rosette fresh weight, floral stalk habitus and phytoplasma titre of healthy and CY-infected Arabidopsis lines

**Fig. 1:**
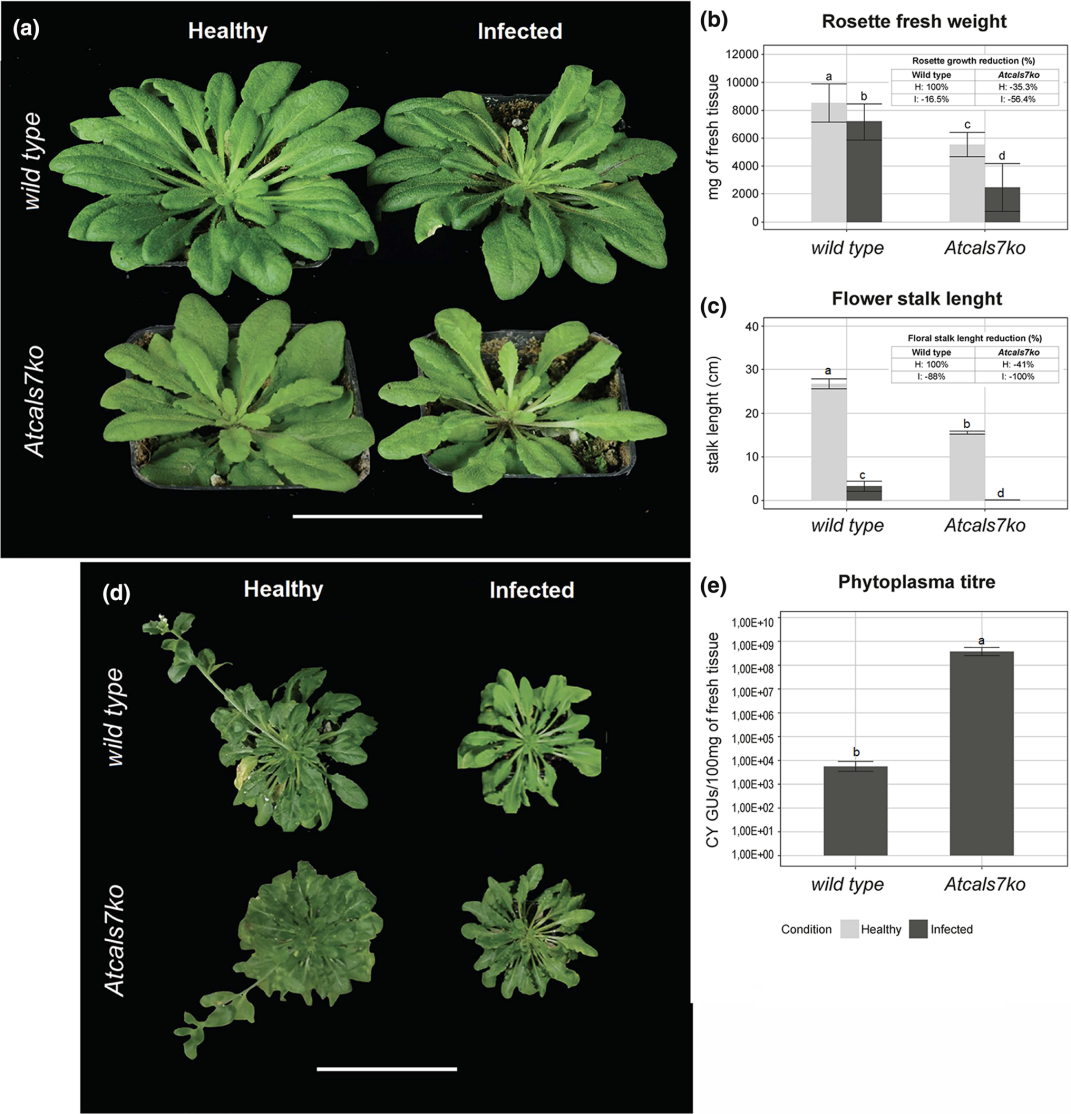
Plant phenotype and phytoplasma titre in wild-type and *Atcals7ko* lines.** a** Representative images of healthy and CY-infected wild-type and *Atcals7ko* plants. Following CY infection, at 20 days after the inoculation access period (IAP), both plant lines showed yellowish small leaves. Leaves having emerged after phytoplasma inoculation were shorter, with thicker main veins and shorter petioles.** b** Fresh weight of rosettes was reduced in *Atcals7ko* plants by 35.3% (inset) as compared to wild-type plants. Following CY infection, rosette fresh weight, on average, was affected by a reduction by 16.5% (inset) in wild-type plants and by 56.4% (inset) in *Atcals7ko* mutants in comparison with their respective healthy controls. **c**, **d** The flower stalk was well developed in healthy wild-type but reduced in length (by 41%, inset) in *Atcals7ko* plants. Following CY infection, the stalk length was strongly reduced (by 88%, inset) in wild-type individuals and absent in the *Atcals7ko* mutants. **e** Phytoplasma titre [expressed as the number of CY-phytoplasma genome units (GUs) per mg of fresh leaf sample] strongly increased in mutant line in comparison to the wild type. In **b** and **c,** statistical analyses were performed using RStudio software Version 1.1.456 (RStudio Team 2020). The normal distribution was checked with the Shapiro–Wilk test. Significant differences among the group means were determined with a two-way ANOVA and post-hoc comparisons between all groups were made with Tukey’s test with *P* < 0.05. In **e**, statistical analysis was performed using RStudio software version 1.1.456 (2009–2018 RStudio). The normal distribution was checked with Shapiro–Wilk test. Significant differences among the means were determined using the Kruskal–Wallis non-parametric test with *P* < 0.05. Error bars indicate standard error of the mean of 8 biological replicates for each condition

A disturbing effect on resource distribution and growth in *Atcals7ko* plants is already known (Barratt et al. 2011; Xie et al. 2011). Therefore, fresh weights of healthy wild type and *Atcals7ko* mutant plants, being an expression of the resource distribution, were quantified first as a control for growth assessment of infected plants. Healthy and CY-phytoplasma-infected wild type and *Atcals7ko* plants were compared as for phenotype, symptom appearance, rosette fresh weight (Fig. 1a, b). Moreover, floral stalk habitus and lengths were also evaluated (Fig. 1c, d). Finally, phytoplasma titre was quantified in infected Arabidopsis lines by qPCR (Fig. 1e).

*Atcals7ko* plants were smaller than wild-type plants, having stunted growth (Fig. 1a) and leaves with a thick main vein. Fresh weight of rosettes (Fig. 1b) was reduced in *Atcals7ko* plants (average weight: 5.5 ± 0.7 g) by 35.3% (inset Fig. 1b) as compared to wild-type plants (average weight: 8.5 ± 1.1 g). Following CY infection, rosette fresh weight, on average, was affected by a reduction by 16.5% (inset Fig. 1b) in wild-type plants (7.1 ± 1.0 g) and by 56.4% (inset Fig. 1b) in *Atcals7ko* mutants (2.4 ± 1.4 g) in comparison with their respective healthy controls. In both lines, leaves that emerged after phytoplasma inoculation were yellowish, small, narrowed, with a thicker main vein and a shorter petiole (Fig. 1a). The mutant line developed a floral stalk reduced by 41% in length (average length: 16.3 ± 0.7 cm) in comparison to the wild type (average length 26.8 ± 2.5 cm) (Fig. 1c, d). CY-infected wild-type plants produced a stalk reduced by 88% (average length 3.3 ± 3.2 cm) compared to the healthy controls, while CY-infected *Atcals7ko* mutants failed to produce a stalk at all (Fig. 1c, d). Finally, phytoplasma titre was significantly higher in mutant plants (*i.e.* 7.6E + 08 phytoplasma genome units (GUs) in 100 mg of leaf sample) than in wild-type plants (*i.e.* 8.2E + 03 phytoplasma GUs in 100 mg of leaf sample) (Fig. 1e).

### Linear speed of phloem translocation

**Fig. 2:**
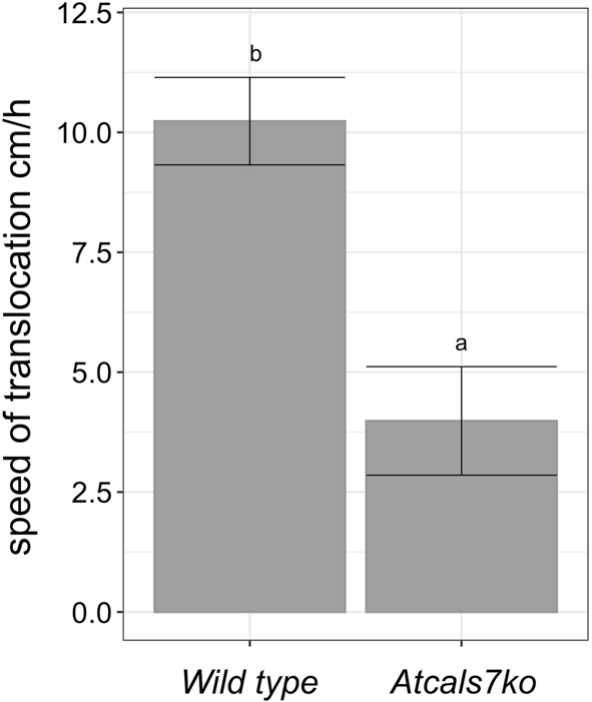
Phloem transport velocity in the flower stalks of wild type and *Atcals7ko* Arabidopsis lines. Carbohydrate translocation velocity along the phloem, measured with ^14^C-labelled photoassimilates. The velocity is calculated by average time of arrival of ^14^C label in the stem phloem tissue near the x-ray detector. Statistical analysis was performed using the Tukey HSD test as the post-hoc test in an one-way ANOVA. Different letters (**a**, **b**) above the bars indicate significant differences, with *P* < 0.05. Error bars indicate the standard error of the mean of 4 biological replicates for each condition

To gain an impression of reduced phloem translocation in *Atcals7ko* plants as a cause of decreased growth (Fig. 1a, b), the speed of longitudinal ^14^C-carbohydrate movement was measured in healthy wild type and *Atcals7ko* Arabidopsis plants (Fig. 2). Linear translocation velocity in flower stalk (expressed as cm h^−1^) was approximatively 50% lower in *Atcals7ko* mutants than in wild-type plants. The average speed in wild-type plants was 10.2 ± 1.6 cm h^−1^, while it was 5.0 ± 2.0 cm h^−1^ in mutants (Fig. 2). As the infected plants developed very short flower stalks (wild type) or even failed to do so (*Atcals7ko*, Fig. 1c, d), it was not possible to determine the translocation speed in CY-infected plants.

### Electron-microscopic observations on midrib vascular bundles

**Fig. 3:**
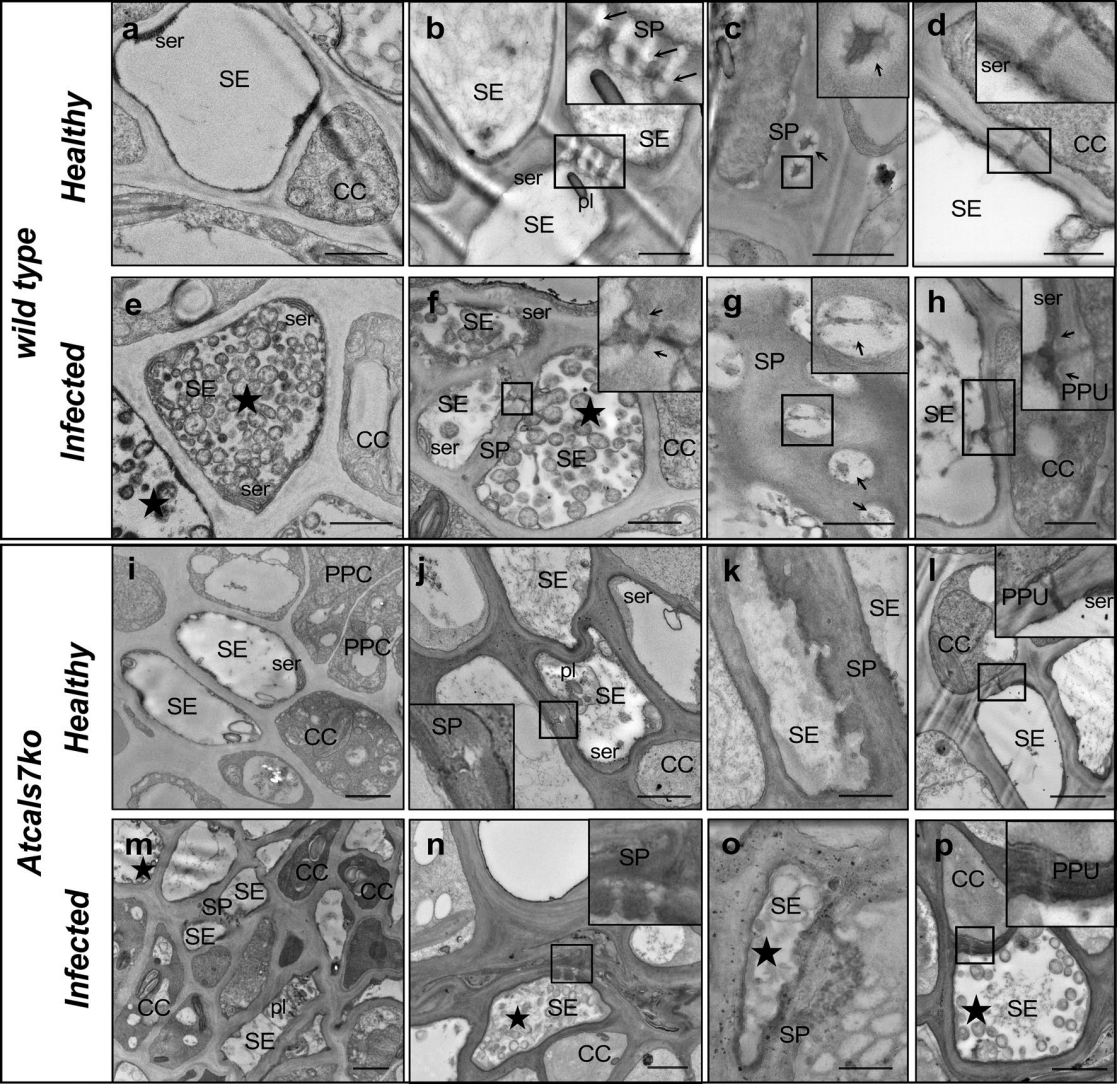
Typical TEM micrographs of phloem tissue in healthy and CY-infected Arabidopsis lines. **a–h** Cross-sections of midribs from healthy (**a–d**) and infected (**e–h**) wild-type Arabidopsis leaves. Healthy samples contain unaltered sieve elements and companion cells (**a**), with a regular shape and no signs of necrosis or subcellular aberrations. The sieve pores, at both lateral (**b**) and ordinary (**c**) sieve plates, are not constricted by callose collars (arrows). Pore-plasmodesma units are open and show their typical shape, unilaterally branched at the CC-side, with sieve-element endoplasmic reticulum located in the proximity of the orifice (**d**). In infected midribs, numerous phytoplasmas are visible inside the sieve elements (**e,** star**)** and at the sieve plate (**f,** star). Lateral sieve plates possess mostly open sieve pores (**f**), whereas in ordinary sieve plates (**g**) pores are constricted by callose depositions (arrows). Pore-plasmodesma units display a similar morphology as in healthy samples, with a thin callose lining at the sieve-element side (**h,** arrows). **I–p** Cross-sections of phloem tissue in midribs of healthy (**i–l**) and CY-infected (**m–p**) *Atcals7ko* Arabidopsis leaves. In healthy *Atcals7ko* samples, phloem cells are apparently well structured (**i**), but the sieve plates show aberrant morphology (**j**, **k**). Sieve pores lack callose and appear not fully developed (**j**, **k**). Pore-plasmodesma units are unilaterally branched, similar to those in wild-type samples (**l**). In CY-infected *Atcals7ko* samples, phytoplasmas (**m**, **n**, **o**, **p**, stars) are visible in sieve elements with thick cell walls (**m, n**). Sieve plates are deformed, thickened (**n**, **o**) and pores are filled by electron-opaque material (**n**). Pore-plasmodesma units are large, without well-defined branches (**p**). In insets, areas of interest of **b**, **c**, **d**, **f**, **g**, **h**, **j**, **l**, **n**, and **p**, are magnified. CC, companion cell; PPC, phloem parenchyma cell; pl, plastid; PPU, pore-plasmodesma unit; SE, sieve element; ser, sieve-element endoplasmic reticulum; SP, sieve plate. Bars correspond to 1 μm

To examine changes in SE ultrastructure in response to pathogen infection, ultrathin sections of midrib vascular bundles were examined under a transmission electron microscope (TEM). Five non-serial sections from 5 healthy or infected plants of both lines (wild type, *Atcals7ko*) were examined. Healthy wild-type samples showed a regular SE and CC ultrastructure (Fig. 3a–d). In lateral (Fig. 3b) and transverse (Fig. 3c) sieve plates, sieve pores were surrounded by a callose lining (Fig. 3b, c) that did not constrict their lumen. At the SE/CC wall interface, sieve-element endoplasmic reticulum (SER) was visible in front of the mouth of the open, typically branched pore-plasmodesma units (PPUs), which are branched at the CC-side (Fig. 3d). In infected wild-type midribs (Fig. 3e–h), numerous phytoplasmas, marked by stars, were visible in the SE lumen (Fig. 3e) or in the proximity of the sieve plates (Fig. 3f). In transverse sieve plates (Fig. 3g), the pores were constricted by callose deposition (Fig. 3g), whereas they appeared mostly open in lateral sieve plates (Fig. 3f). PPUs were open and displayed a slightly thickened callose lining at the SE side (Fig. 3h). As in healthy plants, SER was observed near the PPU mouths in infected plants (Fig. 3h).

In healthy *Atcals7ko* plants (Fig. 3i–l), SEs and CCs appeared identical to those in the wild type (Fig. 3i). Sieve-element protein filaments, plastids and SER were readily recognizable in the SE lumen or close to the plasma membrane (Fig. 3j). In accordance with previous studies (Barratt et al. 2011; Xie et al. 2011), however, sieve plates lacked callose and showed an aberrant morphology (Fig. 3j, k). Some sieve-pore channels seemed to be partially open or not fully developed (Fig. 3j, k), whereas PPUs displayed a normal, one-sided branched appearance (Fig. 3l). Inside SEs of the infected *Atcals7ko* line phytoplasmas were visible (Fig. 3m–p). Many SEs possessed thickened walls (Fig. 3m), while others had collapsed (Fig. 3n). Like in healthy samples, sieve plates appeared to be damaged, with sieve pores filled by electron-opaque material (Fig. 3o). PPUs were enlarged without a well-defined branching (Fig. 3p). In the proximity of PPUs, SER was recognizable in the SEs of both healthy and CY-infected plants (Fig. 3l, p).

### Sugar quantification in midrib tissues

**Fig. 4:**
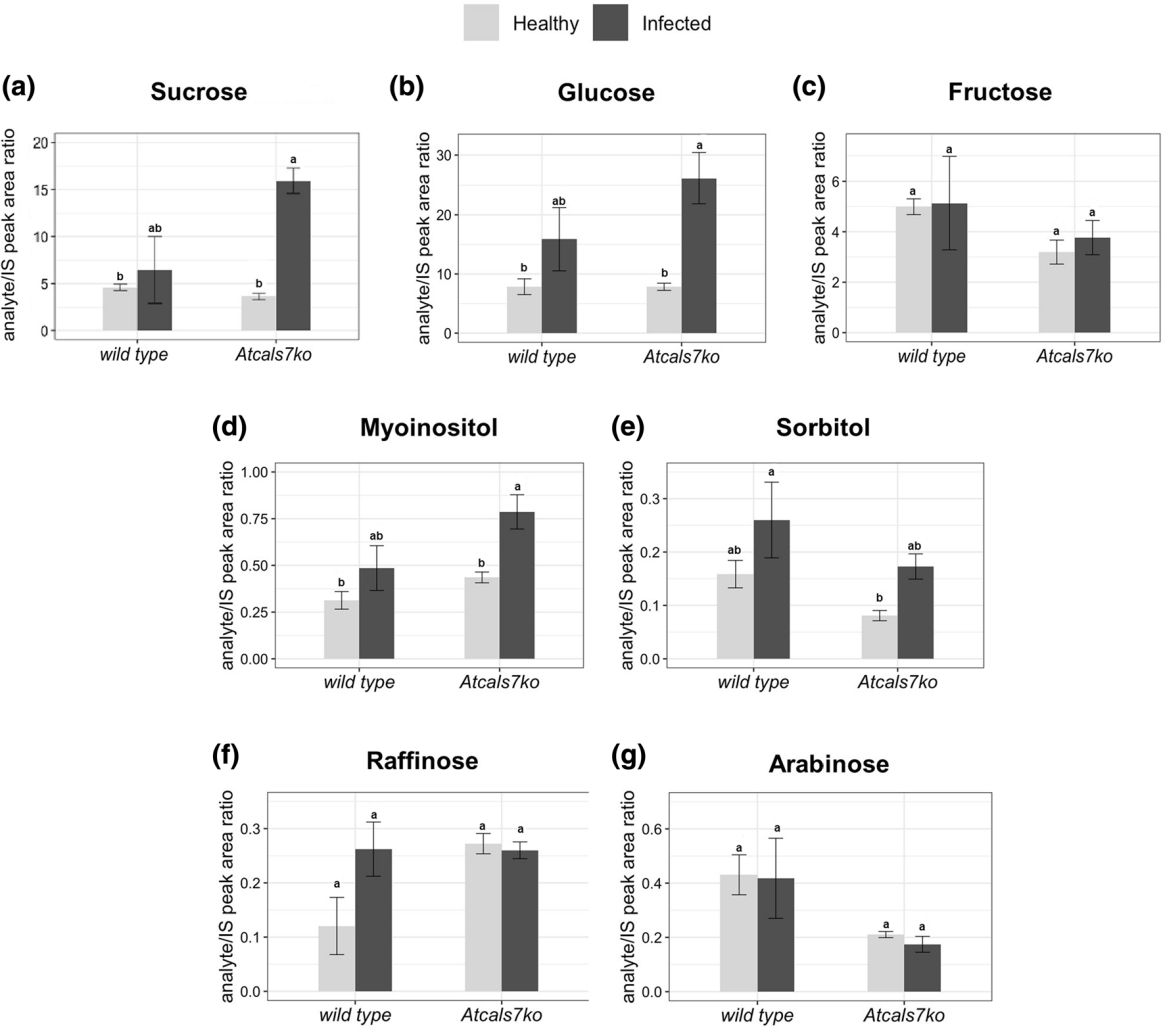
Sugar quantification in the midribs of healthy and infected Arabidopsis lines. Sucrose (**a**), glucose (**b**), fructose (**c**), myo-inositol (**d)**, sorbitol (**e**), raffinose (**f**) and arabinose (**g**) were quantified in midribs of the two different lines, healthy or CY-infected. Data obtained were expressed as analyte/IS peak area ratio. Statistical analysis was performed using the Tukey HSD test as the post-hoc test in a two-way ANOVA. Different letters (**a**, **b**) above the bars indicate significant differences, with *P* < 0.05. Error bars indicate the standard error of the mean of 4 biological replicates for each condition run in triplicate

To identify the impact of the absence of *AtCALS7* and phytoplasma infection on the carbohydrate metabolism, sucrose, glucose, fructose, myo-inositol, sorbitol, arabinose and raffinose contents were quantified in the midribs of healthy and infected wild-type or *Atcals7ko* plants (Fig. 4). No significant differences were found between the non-infected plant lines (Fig. 4a–g). Following CY infection, the amounts of the above-mentioned sugars did not differ significantly from the controls in the wild-type line (Fig. 4a–g). In the *Atcals7ko* line, sucrose, glucose and myo-inositol significantly increased in response to CY infection (Fig. 4a, b, d). As compared to the midribs of healthy *Atcals7ko* plants, sucrose increased approx. fivefold (Fig. 4a), glucose approx. fourfold (Fig. 4b), and myo-inositol approx. twofold (Fig. 4d) in infected *Atcals7ko* plants.

### Gene expression analyses

**Fig. 5:**
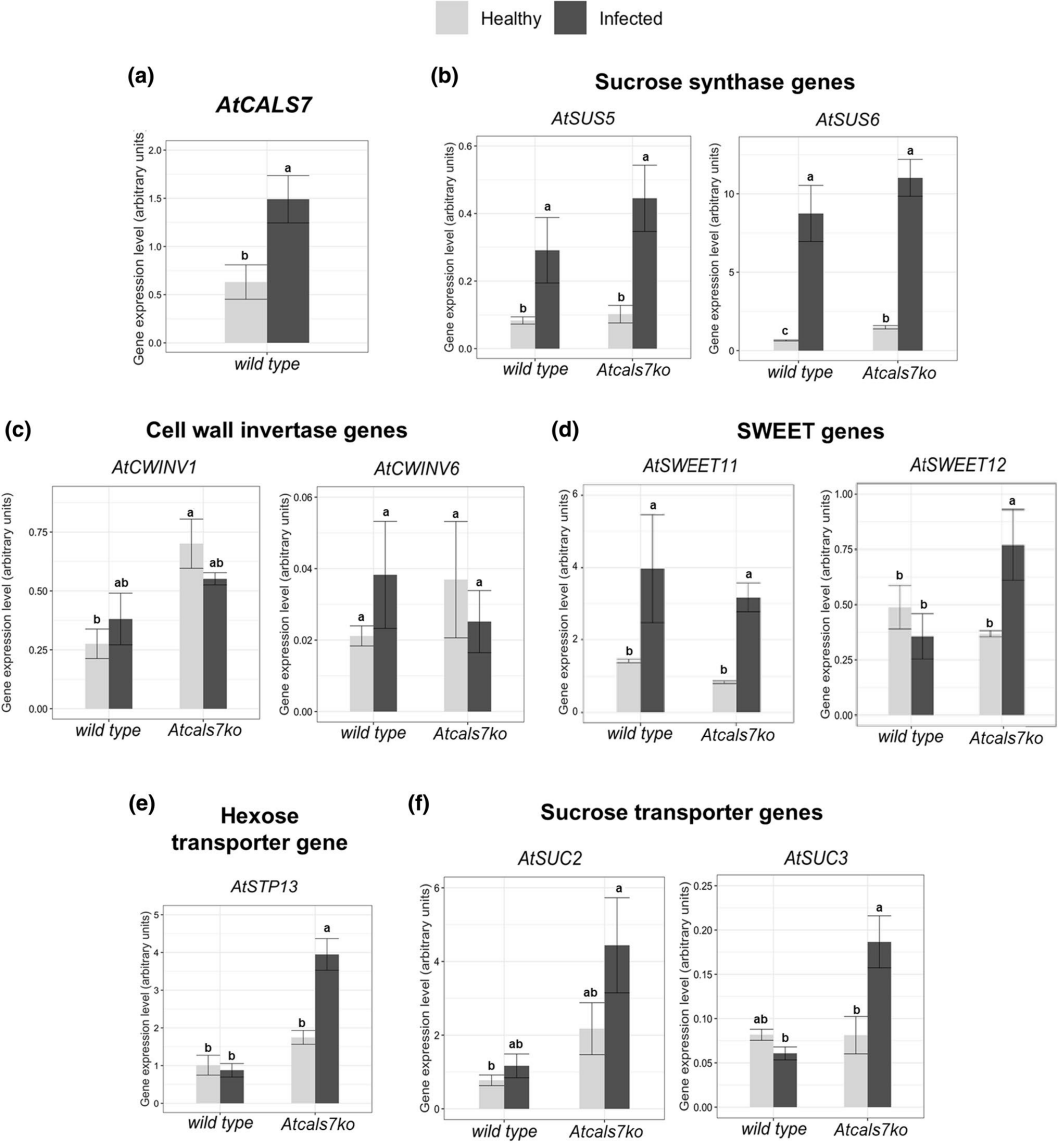
Transcript profiling of genes involved in phloem callose synthesis and sugar transport and metabolism. **a** Expression level of phloem callose synthase gene (*AtCALS7*) in healthy and CY-infected wild-type plants. **b**–**f** Transcript profiling of sucrose synthases AtSUS5 and AtSUS6 (**b**), sucrose transporters AtSUC2 and AtSUC3 (**c**), SWEET sugar facilitators AtSWEET11 and AtSWEET12 (**d**), cell-wall invertases AtCWINV1 and AtCWINV6 (**e**), the hexose transporter AtSTP13 (**f**) in healthy and infected wild type and *Atcals7ko* plants. Healthy and infected plants belonging to the two lines were compared. Expression values were normalized to the *UBC9* transcript level, arbitrarily fixed at 100, then expressed as mean normalized expression ± SD (transcript abundance). Statistical analysis was performed using the Tukey HSD test as the post-hoc test in a two-way ANOVA. Different letters (a, b, c, d) above the bars indicate significant differences, with *P* < 0.05. Error bars indicate the standard error of the mean of 5 biological replicates for each condition run in triplicate

Next, the expression of *AtCALS7* and diverse other genes that are involved in the regulation of carbohydrate household was determined (summarized in Table 1). The expression level of the SE-specific callose synthase 7 gene (*AtCALS7*) was analysed in midribs of healthy and CY-infected wild-type plants and was significantly upregulated (around 2.5-fold) in infected plants (Fig. 5a). Moreover, the expression of genes involved in sugar metabolism and transport of source leaves of Arabidopsis, and known to be localized in the phloem tissue, was investigated. Expression levels of sucrose synthases (*AtSUS5* and *AtSUS6*), sucrose transporters (*AtSUC2* and *AtSUC3*), sugar transport facilitators (*AtSWEET11, AtSWEET12*)*,* vascular high affinity hexose:proton symporter (*AtSTP13*) and cell-wall invertases (*AtCWINV1*, *AtCWINV6*) were determined in the four plant groups under investigation (Fig. 5b–f).

**Table 1:**
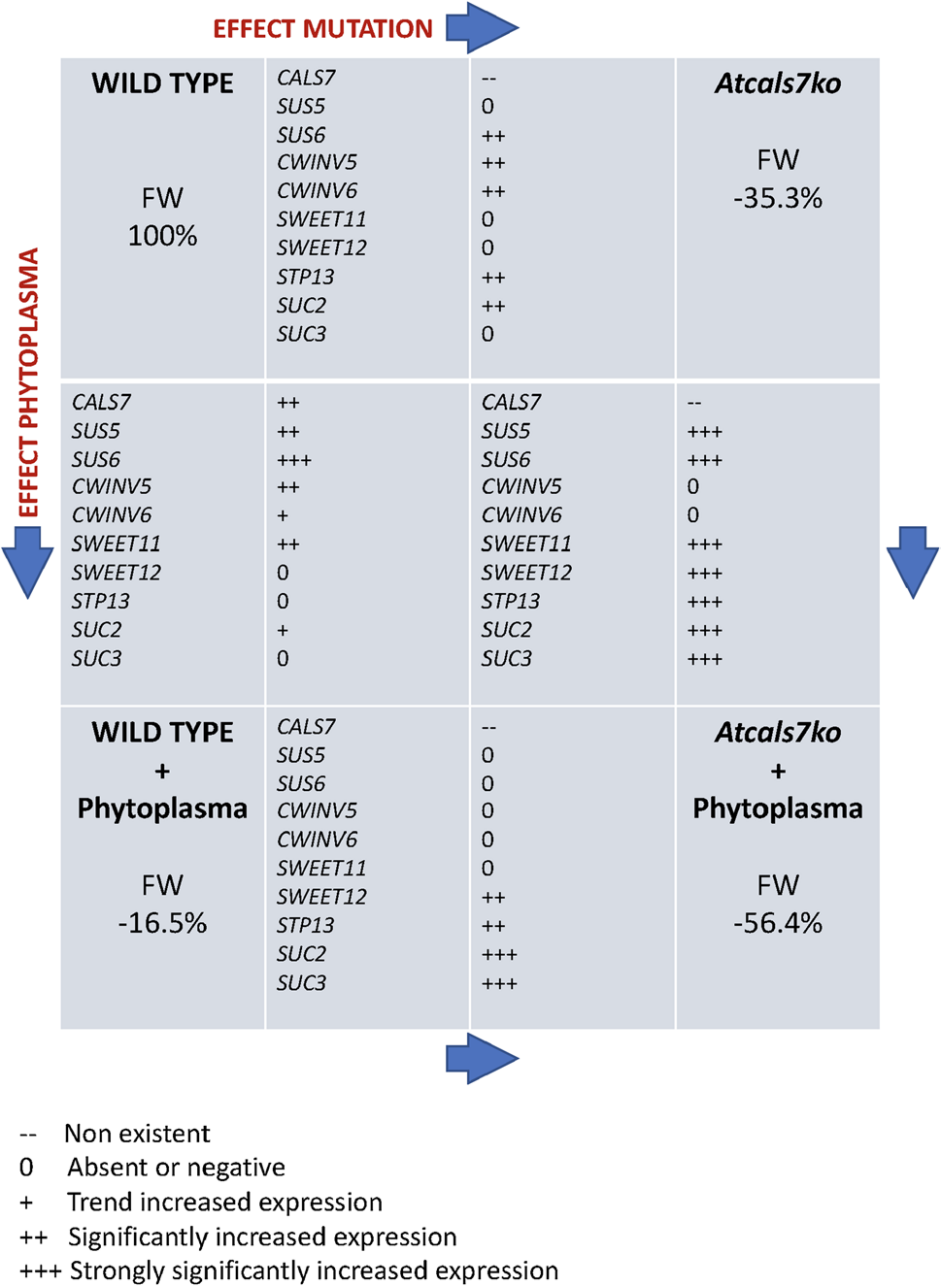
The table sums up the variations of rosette fresh weight and gene expression level in the Arabidopsis lines during CY infection compared to their respective healthy control plants. The data reflect those reported in Figs. 1 and 5

*AtSUS5* and *AtSUS6* encode two sucrose synthases located in the SEs, which provide UDP-glucose as a substrate for AtCALS7 in the callose-synthetizing complex. In comparison with the expression levels in healthy wild-type plants, *AtSUS6* was significantly upregulated in the mutant line (Fig. 5b). *AtSUS5* showed low expression levels which did not differ between the two lines. Following CY infection, AtSUS5 transcripts increased 3.5 and 5 times in wild-type and mutant lines, respectively, while AtSUS6 transcripts increased 13.5 and 7.5 times (Fig. 5b). Cell-wall invertases present a metabolic pathway for extracellular hexose production, as an alternative for the intracellular sucrose synthases. The expression level of *AtCWINV1* was significantly higher in the midrib of healthy *Atcals7ko* plants (Fig. 5c) than in other plant groups. *AtCWINV6* showed a similar trend, but the very low expression level prevented us from calculating a significant difference between the lines (Fig. 5c). In response to infection, the gene encoding the sugar efflux facilitator AtSWEET11 was significantly overexpressed in both Arabidopsis lines (Fig. 5d), while the transcription levels of *AtSWEET12* significantly increased only in *Atcals7ko* line (Fig. 5d). The expression level of *AtSTP13*, was similar in the healthy midribs of both lines, but was enhanced significantly (around 5.5-fold) in the infected midrib of *Atcals7ko* line (Fig. 5e). The transcripts of the two genes *AtSUC2* and *AtSUC3*, encoding sucrose proton symporters, located respectively in CCs and in SEs (Stadler and Sauer 1996; Meyer et al. 2004), were analysed in the midribs of both lines (Fig. 5f). The expression level of *AtSUC2* did not significantly change in all plant groups, while *AtSUC3* was significantly upregulated only in the *Atcals7ko* line following CY infection (Fig. 5f).

All in all, three general trends emerged regarding the gene expression in infected mutant and wild-type plants in comparison with their healthy controls. Modulation was absent for *AtCWINV1* and *AtCWINV6* in both lines, *AtSUS5*, *AtSUS6* and *AtSWEET11* were upregulated in both lines, while *AtSWEET12*, *AtSUC3*, and *AtSTP13* were upregulated in mutants only.

## Discussion

Nutrition of axial sinks is dependent on the action of plasma membrane-located transporters that compete for resources at either side of the apoplasmic gap between SE-CC complexes and parenchyma (Hafke et al. 2005). The competition is regulated by the number of transporters, the inside-outside gradients of resources and the proton-motive forces generated by the cells involved (van Bel 2021). Most of the outward-directed transporters are probably driven by concentration gradients (e.g. SWEETs and Umamits), while most of the inward-directed transporters are fuelled by the motive-motive force (van Bel 2021). The interplay between the transporters along the pathway achieves a flexible release and retrieval resulting in an hop-on hop-off resource traffic in the sieve tubes along the pathway (van Bel 2021; Stadler 2021). Under sink-limiting conditions, however, turgor-effectuated gating of plasmodesmata between SE-CCs and phloem parenchyma cells (PPCs) allows a massive symplasmic efflux towards the axial sinks, which can only be marginally controlled by membrane-bound transporters.

### Interpretation of Arabidopsis growth experiments

The essentials of supply and growth may be used to interpret the present experiments, in which plants are challenged by phytoplasma infection or the absence of *AtCALS7*, which both effect a seriously reduced growth (Fig. 1a, b). Given the fact that both challenges impact on SE biology, it is plausible that severed phloem translocation is a prominent bottleneck in the growth processes here and causes retarded growth. The nature of interference with the supply of terminal sinks and the inherent reduction of growth, however, may be quite different for the absence of *AtCALS7* or phytoplasma infection. An additional complicating factor is that the presence of phytoplasmas activates AtCALS7, possibly due to the rise of the Ca^2+^ concentration in SEs in response to phytoplasma infection (Musetti et al. 2013).

The lack of *AtCALS7* does not only prevent sieve-pore constriction, but also infers an incomplete sieve-pore development (Barratt et al. 2011). In *Atcals7ko* mutants, therefore, longitudinal transport is retarded due to the distorted shape of the sieve pores (Fig. 3k, j; Barratt et al. 2011). Formulae for mass-flow determination (De Schepper et al. 2013 and literature therein) predict that the shape of the sieve plates controls flow velocity. The linear flow velocity of phloem sap as measured by a non-invasive method (Vincent et al. 2019) is halved in comparison to that in wild-type plants (Fig. 2).

The decreased growth of *Atcals7ko* mutants (Fig. 1) indicates that the balance between the supply of terminal and axial sinks is appreciably disturbed. Since carbohydrate concentrations and expression levels of carbohydrate-handling enzymes are similar in wild-type and mutant plants (Figs. 4, 5), the disturbance of resource distribution is likely due to mechanical alterations (e.g. Figure 3). At the same phloem-loading rates, the concentration of carbohydrates must be higher in sieve-tube saps transported at lower velocities. At reduced velocities, moreover, solute retrieval rates by axial sinks along the translocation pathway are expected to be higher in view of the longer residence times of translocate inside the pathway (van Bel 2021). This effect is corroborated by the inability of the PPUs to constrict giving rise to symplasmic release from sieve tubes in mutants. Both factors thus effectuate an increased retrieval by axial sinks, even if their carbohydrate transporters are not upregulated (Supplementary Fig. S1). Knocking out AtCALS7 thus simulates resource outflow into axial sinks, whereas nutrient supply to terminal sinks is restricted leading to a massive growth reduction to 56% (Fig. 1b; Table 1). An illustration of a diminished transport to terminal sinks in mutants is provided by the length of the flower stalks, which showed a 41% length reduction (Fig. 1c). The effect of phytoplasma infection on plant growth is less dramatic than that of the absence of AtCALS7 (Fig. 1b). Despite the presence of phytoplasmas as intracellular sinks in sieve elements of wild-type plants, withdrawal of carbohydrates from the phloem stream along the pathway seems to be less than in mutants, since growth is reduced to only 84% (Fig. 1b).

It is puzzling, how infected wild-type plants appear to succeed a better maintenance of supply of the terminal sinks than *Atcals7ko* plants. A reason may be that callose does not constrict all sieve pores (Gallinger et al. 2021; Buoso et al. 2022), but stem stunting (Pagliari et al. 2016) is indicative of a serious disturbance of phloem function (Maust et al. 2003). Perhaps, constriction of sieve pores—including those of PPUs—due to phytoplasma infection prevents lateral symplasmic loss from SEs and loss is solely controlled by membrane-located release/retrieval transporters (van Bel 2021). The latter are more effective in retrieving resources by SEs than in mutant plants, where resources are presumably released symplasmically from sieve tubes without intervention of membrane-located transporters. It seems beyond doubt that phytoplasmas exploit the absence of AtCALS7 for better recruitment of resources for growth and propagation. Remarkably, floral stalk length is less reduced in mutants than in infected plants (Fig. 1c, d), whereas the growth rates shows the opposite proportion (Fig. 1c). This apparent contradiction probably demonstrates that there are unknown players in the game than transporter-mediated distribution alone. The exceptional growth reduction of infected mutants (Fig. 1) rises the suspicion that the effects of AtCALS7 absence and phytoplasma infection are synergistic rather than additive, which indeed speaks for the involvement of yet unknown factors.

There are a few speculative reasons for the higher phytoplasma titre in *Atcals7ko* mutants than in wild-type plants (Fig. 1d). As a first possibility, the disability to sieve-pore constriction allows a wider spread of the phytoplasmas. Another possibility is that sieve-pore (and perhaps plasmodesmal) corridors are not constricted or have even been widened so that phytoplasma effectors can move more easily and enable a better recruitment of resources.

### Phytoplasma infection increases the carbohydrate content in midribs of *Atcals7ko* plants

Midribs of healthy source leaves possess similar carbohydrate levels in the wild-type and *Atcals7ko* lines (Fig. 4). Production of sucrose and its metabolites is not influenced by the loss of AtCALS7 (Fig. 4). An enhanced carbohydrate metabolism in response to phytoplasma infection was derived from increased glucose, sucrose and myo-inositol levels in midribs of mutant and wild-type plants (Yao et al. 2019). Such an upregulation is more significant in mutant than in wild-type plants (Fig. 4). The higher concentration and the upregulation of AtSUS5 and AtSUS6 may reflect the high need of phytoplasmas for ready high-energy oligosaccharides (Yao et al. 2019, 2020). In the infected mutant line, a high amount of myo-inositol was detected. In the absence of callose, myo-inositol could be a useful substitute to modulate SE cell-wall plasticity as a response to stress imposed by phytoplasmas as demonstrated for other environmental cues (Wu et al. 2018). Myo-inositol is an excellent carbon source for cell-wall constituents such as pectin and hemicellulose (Loewus and Murthy 2000), which are together with cellulose the major polysaccharides in Arabidopsis cell walls (Bethke et al. 2016).

It should be noted that modified carbohydrate concentrations under various conditions are hard to interpret, even if the differences are significant in a number of instances (glucose, sucrose, myo-inositol; Fig. 4). Because it is unknown, which cell types or cell compartments are responsible for the measured contents of entire tissues, assessment of the mechanisms involved remains beyond speculation.

### The release/retrieval model and expression of genes involved in carbohydrate processing

As expected (e.g. Barratt et al. 2011), the expression of *AtCALS7* is strongly enhanced by phytoplasma infection in wild-type plants (Fig. 5; Table 1), since CY-infected plants show thick callose collars around the sieve pores (Pagliari et al. 2017). In keeping with the increased production of callose, the expression of the genes *AtSUS5* and *AtSUS6* which are united in a callose-spinning apparatus is upregulated (Fig. 5; Table 1). In this way, precursors are provided for callose synthesis (Tan et al. 2015; De Marco et al. 2021) and for the synthesis of carbon skeletons of defence-related compounds (Bolouri-Moghaddam et al. 2010; Musetti et al. 2013). For logical reasons phytoplasmas fail to induce a higher expression level of the above genes in *Atcals7ko* plants (Table 1, lower panel) in view of the absence of a callose-synthetizing enzyme in the SE membrane. By contrast, it is unclear why *AtCWInv1* and *AtCWInv6* are only upregulated to some degree in infected wild-type plants (Fig. 5); apoplasmic breakdown of sucrose may be of marginal importance for the provision of monosaccharides.

**Fig. 6:**
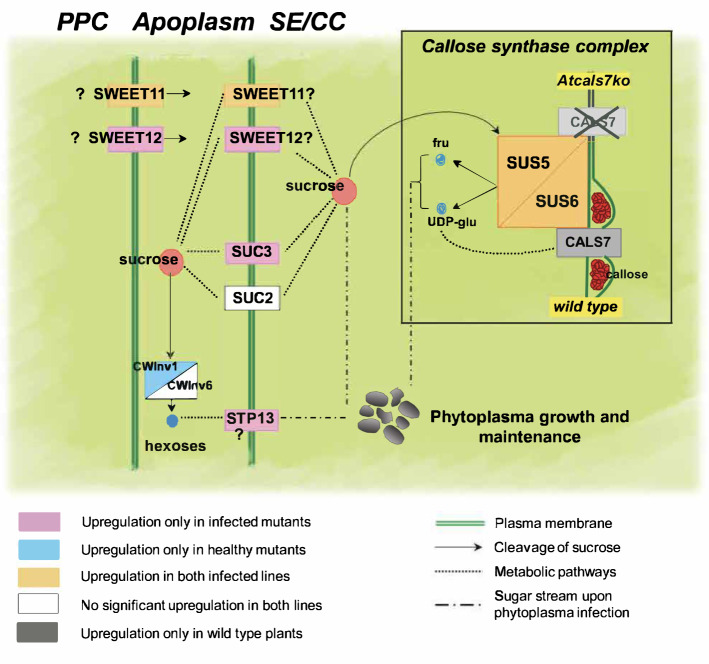
Localization of proteins involved in sugar metabolism and transport in the midribs of wild type and *Atcals7ko* Arabidopsis and their expression in response to phytoplasma infection. In transport phloem, AtSUC2, AtSUC3, are localized to the plasma membrane of SE/CC complexes (Meyer et al. 2004; Gould et al. 2012). AtSTP13 is reported to be localized in the phloem, without specification about the type of cell (Yamada et al. 2011). In the model we are proposing, we located AtSTP13 in the SE/CC complex, as best in line with the results, without giving an absolute indication. Deployment of SWEET11 and SWEET12 is less certain, as they were localized to the phloem parenchyma (Le Hir et al. 2015) but also to the companion cells (Abelenda et al. 2019). AtSUS5 and 6 are localized to the SE plasma membrane. Synthesis of callose requires sucrose units that are cleaved by sucrose synthases into fructose and UDP-glucose, the substrate for callose synthase. AtSUS5 and AtSUS6 associated with AtCALS7 form a unique enzyme complex (Bieniawska et al. 2007; Ruan 2014; Stein and Granot 2019). Independently from the different protein localization, and regardless of the carbohydrate form (sucrose or hexoses), upregulation of *AtSUC3*, *AtSTP13* and *AtSWEET12* in *Atcals7ko* plants would stimulate sugar transport to SE/CC complexes (dotted lines), supporting phytoplasma maintenance (dashed lines) and giving rise to an increased susceptibility of the mutant line to phytoplasma infection

According to the release/retrieval concept as the basis for resource distribution over the sinks, both *Atcals7ko* mutants and infected plants are expected to compensate resource loss along the pathway by upregulation of retrieval. In this way, plants will try to restore their “grip” on the resources by upregulated expression of genes involved in retrieval by SEs. The apparent resource loss along the phloem pathway is higher in *Atcals7ko* plants than in infected wild-type plants (Fig. 1b). The urge to counter the massive loss in sugars may be mirrored by the upregulation of *AtSTP13* and *AtSUC3*, (Table 1, upper panel; Fig. 6), whereas the upregulation of these genes is virtually absent in infected wild-type plants (Table 1, left panel). There, *AtSUC2*, *AtSUC3* and *AtSTP13* are not upregulated at all. AtSUC2 and AtSUC3 are not only responsible for phloem loading, but are also engaged in sucrose retrieval along the transport pathway (Meyer et al. 2004; Gould et al. 2012). *AtSTP13* belongs to a gene family encoding for hexose cotransporters and is strongly expressed in the leaf veins (Yamada et al. 2011). It contributes to the basal resistance to necrotrophic fungi (i.e. *Botrytis cinerea*, Lemonnier et al. 2014) and extracellular bacteria (i.e. *Pseudomonas syringae*, Nørholm et al. 2006) by withdrawing apoplasmic sugars. ABA and the bacterial peptide flg22 were shown to induce *AtSTP13* (Yamada et al. 2016), probably mediated by the transcription factor MYB96, which binds STP13 promoter and induces gene expression upon exogenous application of ABA and fgl22 (Lee and Seo 2021). By contrast, overexpression of *STP13* in plants infected with intracellular biotrophic pathogens provokes increased host susceptibility by promoting cytoplasmic hexose accumulation (Huai et al. 2020; Skoppek et al. 2022). As phytoplasmas are biotrophic intracellular pathogens, it is quite conceivable that overexpression of *STP13* in CY-infected *Atcals7ko* plants is indicative for an increased susceptibility to phytoplasma attack in comparison to the wild-type (Berzrutczyk et al. 2018b). As a matter of fact, the mutant appears more affected by infection (i.e. Fig. 1). Comparison of the expression levels shows that mainly transporter genes, located in the plasma membrane of phloem parenchyma cells and SE-CC complexes, are overexpressed in infected plants (Table 1, lower panel), which is a clear difference with the genes overexpressed in the absence of AtCALS7 (Table 1, upper panel). Thus phytoplasmas induce the expression of transporters for sugar retrieval (*AtSTP13*; *AtSUC2*, *AtSUC3;* Fig. 6). The apparent corroborative effects of the lack of AtCALS7 and phytoplasmas render the overexpression of these transporter genes even more pregnant (Table 1, right panel). Thus, both modifications seem to elicit upregulation by oligosaccharide transporter genes and, hence, carbohydrate retrieval by transporters to fuel pathogen proliferation (Veillet et al. 2017). In this context, it is worth noting that both host and phytoplasma have shared interest and therefore logically cooperate in sugar retrieval by SEs.

AtSWEET 11 and 12 were characterized as sucrose effluxers localized to the plasma membrane of phloem parenchyma cells (PPCs) that release photoassimilates into the apoplasmic space around the SEs (Chen et al. 2012). They are expressed in most Arabidopsis tissues (Braun 2012) and therefore play various roles (Breia et al. 2021). OsSWEETs are tentatively localized to phloem parenchyma in the phloem-unloading zone of rice (Milne et al. 2018), and StSWEET11 was proposed to be deployed in the plasma membrane of the companion cells of potato (Abelenda et al. 2019). In the frame of the present study, the occurrence of SWEET11 and 12 in the floral stalks of Arabidopsis is meaningful (Le Hir et al. 2015). With the present arguments at hand, it is not excluded that SWEET11 and SWEET12 are located in the plasma membrane of SE-CC complexes and phloem parenchyma. Because their cellular location in transport phloem is uncertain as yet, the significance of the upregulation of *AtSWEET11* and *AtSWEET12* is unclear (Fig. 6). Increased sucrose release from phloem parenchyma cells (PPCs) into the apoplasmic space would make carbohydrates readily available for intensified retrieval by SEs. The possibility that SWEETs determine plant susceptibility or resistance by controlling the nutrient supply to pathogens has recently been discussed (Chen et al. 2010; Bezrutczyk et al. 2018a; Breia et al. 2021). *AtSWEET11* and *12* are members of clade III of the SWEET genes, which are involved in disease development (Li et al. 2018). It has been reported that OsSWEET12 (Li et al. 2013) and AtSWEET11 (Wipf et al. 2021) interact with AtRBOHD, a membrane NADP oxidase producing reactive oxygen species involved in defence-related processes. In line with the present interpretation, SWEETs were proposed to modulate the sucrose availability for pathogens (Fatima and Senthil-Kumar 2021).

## Concluding remarks

It appears that the release-retrieval model provides a useful basis for a provisional explanation of the present results. As sugar transporters are regulated not only at transcriptional, but also at post-transcriptional and at post-translational levels (Liesche et al. 2011; Wipf et al. 2021; Garg and Kühn 2022), many additional molecular factors might participate in their modulation in Arabidopsis following phytoplasma infection, including the possibility of protein redistribution to regulate membrane permeability. Moreover, plasmodesmal connectivity is worthy to be considered, as it determines if and how far the phytoplasma effectors can disseminate into the tissues surrounding the sieve tubes and control the recruitment of nutrients. These should be therefore some of the focus issues of further research.

### *Author contribution statement*

RM conceived the project. CB and GM grew the plants, reared the insects and prepared inoculum sources. CB, SB and SS performed the molecular analyses. AmL conceived sugar quantification and CV the flow speed experiment. CB, JHS and YW performed the sugar quantification assays. RM and AJEvB wrote the manuscript with the valuable support of SS. All the authors provided critical suggestions for the realization of the manuscript.

## Supplementary Information

Below is the link to the electronic supplementary material.

Supplementary file1 Hypothetical model for the impact of functional (wild-type line) or aberrant (*Atcals7ko* line) sieve plates on the photo-assimilate investment into terminal or axial sinks. The red arrowheads quantify the extent of investment in terminal sinks, which is correlated to the translocation speed. The size of the horizontal black arrows quantifies photoassimilates which are invested in axial sinks. Photoassimilate investment into terminal sinks is lower in the *Atcals7ko* line, due to aberrant sieve plates, which results in more escape of carbohydrates along the pathway towards the axial sinks. In case of phytoplasma infection, photo-assimilate investment into terminal sinks could be more affected, favouring not only the axial sink proliferation, but also the phytoplasma (additional sink) nourishment (DOCX 2248 KB)

Supplementary file2 Primers used for gene expression analysis (DOCX 15 KB)

Supplementary file3 Figure (**a**) and Movie (**b**) showing the apparatus used for the measurement of the phloem transport linear speed. Detector 1 acts as control: it allows to evaluate whether C14 is inside the plant after the labelling. Detectors 2 and 3 are used for the data collection (PPTX 6909 KB)

## Data Availability

The data that support the findings of this study are openly available in BioRxiv repository at https://doi.org/10.1101/2021.06.25.449948.

## Abbreviations

CALS7: Callose synthase 7
CC: Companion cell
CWINV: Cell-wall invertase
PPU: Pore-plasmodesma unit
SE: Sieve element
STP: Sugar transporter protein
SUC: Sucrose transporter
SUS: Sucrose synthase
SWEET: Sugar will eventually be exported transporter
