# Supplementary material for: Increased susceptibility to Chrysanthemum Yellows phytoplasma infection in Atcals7ko plants is accompanied by enhanced expression of carbohydrate transporters
Source: Planta. 2022 Jul 17;256(2):43. doi: 10.1007/s00425-022-03954-8 (PMC9288947; doi:10.1007/s00425-022-03954-8)
Supplement: Supplementary file 1 — Supplementary file1 Hypothetical model for the impact of functional (wild-type line) or aberrant (Atcals7ko line) sieve plates on the photo-assimilate investment into terminal or axial sinks. The red arrowheads quantify the extent of investment in terminal sinks, which is correlated to the translocation speed. The size of the horizontal black arrows quantifies photoassimilates which are invested in axial sinks. Photoassimilate investment into terminal sinks is lower in the Atcals7ko line, due to aberrant sieve plates, which results in more escape of carbohydrates along the pathway towards the axial sinks. In case of phytoplasma infection, photo-assimilate investment into terminal sinks could be more affected, favouring not only the axial sink proliferation, but also the phytoplasma (additional sink) nourishment (DOCX 2248 KB) [file 425_2022_3954_MOESM1_ESM.docx]

**Supplementary Fig. S1** Hypothetical model for the impact of functional (wild-type line) or aberrant (*Atcals7ko* line) sieve plates on the photoassimilate investment into terminal or axial sinks. The red arrowheads quantify the extent of investment in terminal sinks, which is correlated to the translocation speed. The size of the horizontal black arrows quantifies photoassimilates which are invested in axial sinks. Photoassimilate investment into terminal sinks is lower in the *Atcals7ko* line, due to aberrant sieve plates, which results in more escape of carbohydrates along the pathway towards the axial sinks. In case of phytoplasma infection, photoassimilate investment into terminal sinks could be more affected, favoring not only the axial sink proliferation, but also the phytoplasma (additional sink) nourishment.
