# Supplementary material for: Increased susceptibility to Chrysanthemum Yellows phytoplasma infection in Atcals7ko plants is accompanied by enhanced expression of carbohydrate transporters
Source: Planta. 2022 Jul 17;256(2):43. doi: 10.1007/s00425-022-03954-8 (PMC9288947; doi:10.1007/s00425-022-03954-8)
Supplement: Supplementary file 2 — Supplementary file2 Primers used for gene expression analysis (DOCX 15 KB) [file 425_2022_3954_MOESM2_ESM.docx]

**Supplementary Table S1** Primers used for gene expression analysis.

| **Gene** | **Locus** | **Forward**  **(sequence 5’-3’)** | **Reverse**  **(sequence 5’-3’)** | **Accession NCBI** |
| --- | --- | --- | --- | --- |
| ***AtCALS7*** | At1g06490 | TTATTTGTCGTCTGCGGCCT | TCCTTTGAACACGCTACGCA | NM_100528.2 |
| ***AtSUS5*** | At5g37180 | TGGAAGCAAAGAGAGGGCTG | CCAGGAGCTGCGATGTTGAA | NM_123077.2 |
| ***AtSUS6*** | At1g73370 | ACATAGCCAAGGAGCTTCGC | CTTGAGCCGAGTTAGCACCA | NM_001198461.1 |
| ***AtCWINV1*** | At3g13790 | ACTCGGCTAAGAACCGGAGA | CGGACCAGCCTTTCTCAACA | NM_001338080.1 |
| ***AtCWINV6*** | At5g11920 | GTCACAGTTGTTGCCGAACC | GGCGGAACCATCACAGGATT | NM_001343222.1 |
| ***AtSUC2*** | At1g22710 | GTCGCTGGAGCTGGTTTAGT | TATCGCTATGGCTCGCGTTT | NM_102118.4 |
| ***AtSUC3*** | At2g02860 | TGTTCTGCTTGTGGATGGCT | ACATGCAGCACAACATGCTC | NM_201675.2 |
| ***AtSWEET11*** | At3g48740 | AGGCACAGTTTCATCCCCTG | TGCTTGCCATGTTTAGGGGT | NM_114733.4 |
| ***AtSWEET12*** | At5g23660 | CCCGGAACCAAAGATCGACA | GCACGGGAGAGAGGAAAACA | NM_122271.3 |
| ***AtSTP13*** | At5g26340 | ATAGGTGTGGCTCTCAACGC | GACAAGAACAACGGAACGGC | NM_122535.4 |
