# Supplementary material for: Increased susceptibility to Chrysanthemum Yellows phytoplasma infection in Atcals7ko plants is accompanied by enhanced expression of carbohydrate transporters
Source: Planta. 2022 Jul 17;256(2):43. doi: 10.1007/s00425-022-03954-8 (PMC9288947; doi:10.1007/s00425-022-03954-8)
Supplement: Supplementary file 3 — Supplementary file3 Figure (a) and Movie (b) showing the apparatus used for the measurement of the phloem transport linear speed. Detector 1 acts as control: it allows to evaluate whether C14 is inside the plant after the labelling. Detectors 2 and 3 are used for the data collection (PPTX 6909 KB) [file 425_2022_3954_MOESM3_ESM.pptx]

## Slide 1
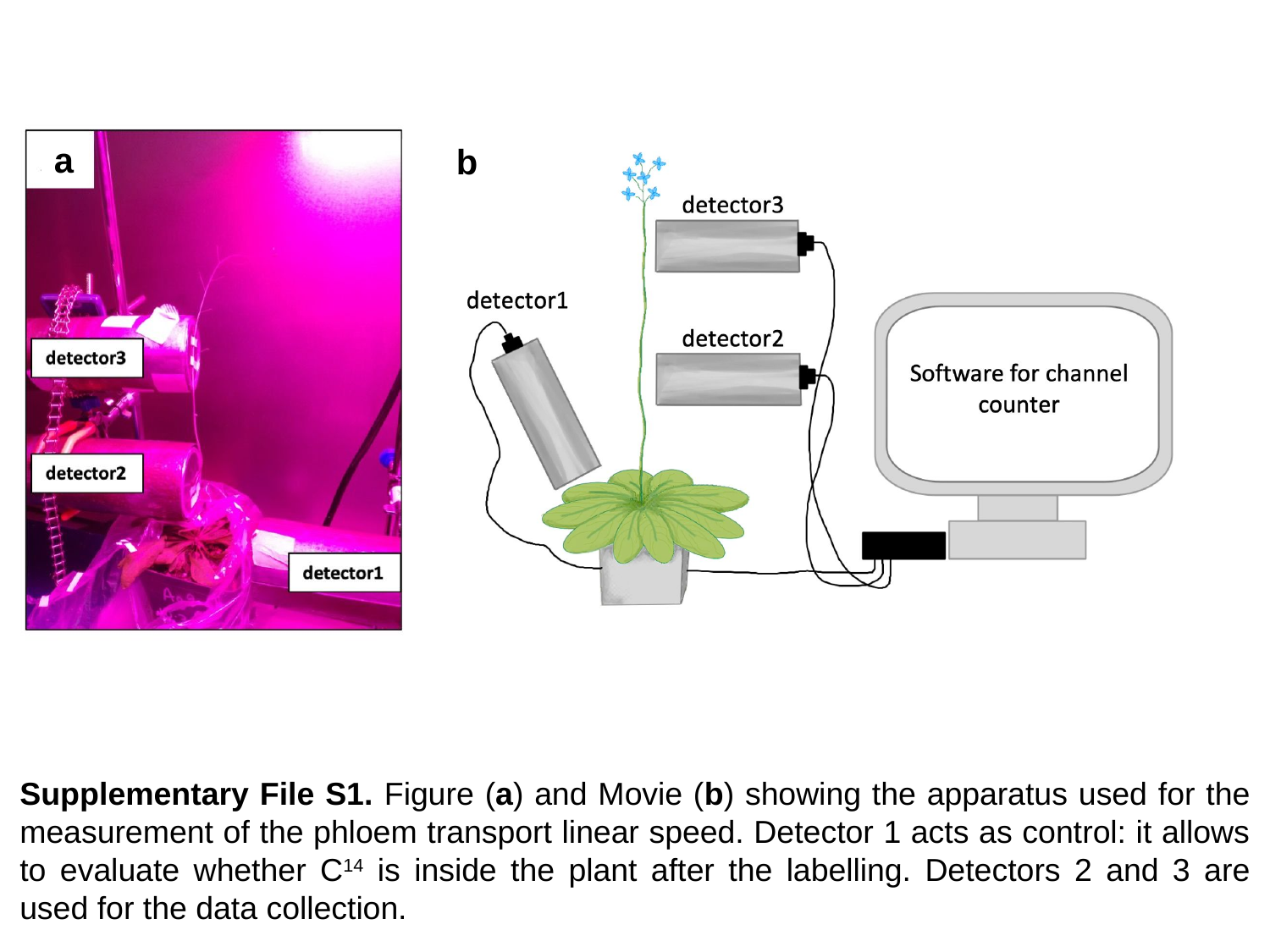

a
b
Supplementary File S1. Figure (a) and Movie (b) showing the apparatus used for the measurement of the phloem transport linear speed. Detector 1 acts as control: it allows to evaluate whether C14 is inside the plant after the labelling. Detectors 2 and 3 are used for the data collection.
